# *Isisfordia molnari* sp. nov*.*, a new basal eusuchian from the mid-Cretaceous of Lightning Ridge, Australia

**DOI:** 10.7717/peerj.7166

**Published:** 2019-06-21

**Authors:** Lachlan J. Hart, Phil R. Bell, Elizabeth T. Smith, Steven W. Salisbury

**Affiliations:** 1 Palaeoscience Research Centre, School of Environmental and Rural Science, University of New England, Armidale, New South Wales, Australia; 2 Australian Opal Centre, Lightning Ridge, New South Wales, Australia; 3 School of Biological Sciences, The University of Queensland, Brisbane, Queensland, Australia

**Keywords:** Isisfordia, Crocodyliformes, Australia, Cretaceous, Eusuchia, Griman Creek Formation, Gondwana, Lightning Ridge, Winton Formation, Isisford

## Abstract

The Australian Mesozoic crocodyliform record is sparse in comparison to other Gondwanan localities. A single formally-named taxon is known from this interval; *Isisfordia duncani* (Winton Formation, Albian–Turonian, Queensland). We present a previously undescribed crocodyliform braincase from the Griman Creek Formation (Cenomanian), New South Wales, which we assign to *Isisfordia molnari* sp. nov. Assignment to the genus is based on the possession of a newly-defined autapomorphy of *Isisfordia*: a broadly exposed prootic within the supratemporal foramen. A second autapomorphy of *I. duncani* (maximum diameter of the caudal aperture of the cranioquadrate siphonium approximately one-third the mediolateral width of the foramen magnum, with the lateral wall of the caudal aperture formed exclusively by the quadrate) may also be present in *I. molnari*; however, definitive recognition of this feature is marred by incomplete preservation. The new taxon is differentiated from *I. duncani* based on the absence of a median ridge on the parietal, and the lack of characteristic ridges on the parietal that form the medial margin of the supratemporal foramina. Reanalysis of a second specimen (the former holotype of the nomen dubium,*‘Crocodylus (Bottosaurus) selaslophensis’*) allows for its referral to the genus *Isisfordia*. Crucial to this reappraisal is the reinterpretation of the specimen as a partial maxilla, not the dentary as previously thought. This maxillary fragment possesses specific characteristics shared only with *I. duncani*; namely an alveolar groove. However, several key features differentiate the maxillary fragment from *I. duncani*, specifically the presence of continuous alveolar septa, the thickening of the medial alveolar rim, and the alveolar and crown base morphology. These findings constitute the first evidence of *Isisfordia* outside of the type locality and indicate its widespread occurrence on the freshwater floodplains along the eastern margin of the epeiric Eromanga Sea during the Albian–Cenomanian.

## Introduction

The crocodyliform taxon, ‘*Crocodilus (?Botosaurus) selaslophensis*’, has remained enigmatic for over 100 years since its discovery (Etheridge, 1917). The name-bearing specimen, a jaw fragment, (AM F15818) comes from the Cenomanian (Bell et al., 2019) Griman Creek Formation, near the Australian opal-mining town of Lightning Ridge, in central northern New South Wales, and was the first fossil crocodyliform described from Australia. Etheridge’s (1917) assignment (and misspelling of both generic names) of the specimen stood unattested until a reanalysis by Molnar (1980) found that it possessed features—primarily an alveolar groove—that indicated there was no reason to refer it to either *Crocodylus* or *Bottosaurus* (spellings amended by Molnar, 1980). Pending the discovery of additional material, Molnar (1980) proposed that the species should be considered indeterminate. Additional crocodilian remains from Lightning Ridge described by Molnar (1980) included a fragment of right maxilla (AM F18628), two procoelous cervical vertebrae (QM F9507 and F10240) and a cervical neural arch (AM F60081), a sacral vertebra (AM F185819), a cranial caudal vertebra (AM F60080), a cervical rib (AM F60082), an incomplete distal femur (in the private collection of K. Barlow) and two left tibiae (AM F18630 and F15821). Molnar (1980) was of the opinion that all this material probably belonged to ‘*Crocodylus selaslophensis’*, and possibly the same individual (based on the matching size of the specimens) but, given that no two specimens were found in association, refrained from formally assigning them. This material is currently under study by the first author (LJH) and will be presented elsewhere.

Molnar & Willis (2001) described additional crocodyliform material from Lightning Ridge, comprising of two maxillary fragments and two dentary fragments. These pieces (the originals of which cannot be currently located) were hypothesised to belong to a taxon distinct from *‘C. selaslophensis’*, based on the lack of an alveolar groove in this new material, and apparent ziphodonty of the teeth. Molnar & Willis (2001) subsequently refuted the suggestion initially forwarded by Molnar (1980) that all Lighting Ridge crocodyliform material pertains to the same taxon but could not advance beyond this suggestion due to the absence of overlapping material. Recently, Mannion et al. (2015; supp. info.) considered *‘C. selaslophensis’* a *nomen dubium*, based on lack of “taxonomic opinion data” (pg. 9). We interpret this phrase as referring to the fragmentary nature of the holotype of *‘C. selaslophensis’* which does not hold enough characters to be taxonomically informative (but see descriptions and discussion below). We reaffirm this assignment, and formally recognise *‘C. selaslophensis’* as a *nomen dubium*.

Etheridge (1917) initially described AM F15818 as a fragment of dentary with six *in situ* teeth. This interpretation was repeated by most subsequent authors (Molnar, 1980; Molnar & Willis, 2001; Kear & Hamilton-Bruce, 2011). Conversely, Smith (1999) considered the fragment as a portion of the maxilla, which we accept as the correct interpretation (see below).

Prior to the description of *Isisfordia duncani* by Salisbury et al. (2006), ‘*C. selaslophensis*’ was the only formally described crocodyliform taxon from the Mesozoic of Australia, and the material it was based on was among the first fossils to be described from the Griman Creek Formation (Etheridge, 1917). Apart from fragmentary material from the Otway Basin in Victoria (provisionally referred to Susisuchidae by Salisbury et al., 2003; see also Poropat et al., 2018), the Griman Creek and Winton formations are the only Australian Cretaceous formations to yield significant Mesozoic crocodyliform remains (Molnar, 1980; Willis, 1997; Molnar & Willis, 2001; Salisbury et al., 2006; Hocknull et al., 2009; Syme et al., 2016).

Here, we reassess the identity of the Griman Creek Formation crocodyliform(s) based on a newly-identified partial braincase and a re-evaluation of the ‘*C. selaslophensis’* holotype, which has implications for the mid-Cretaceous crocodyliform diversity and distribution along the eastern margin of the Eromanga Sea.

## Materials & Methods

The electronic version of this article in Portable Document Format (PDF) will represent a published work according to the International Commission on Zoological Nomenclature (ICZN), and hence the new names contained in the electronic version are effectively published under that Code from the electronic edition alone. This published work and the nomenclatural acts it contains have been registered in ZooBank, the online registration system for the ICZN. The ZooBank LSIDs (Life Science Identifiers) can be resolved and the associated information viewed through any standard web browser by appending the LSID to the prefix  http://zoobank.org/. The LSID for this publication is: *urn:lsid:zoobank.org:pub:C38C6FDF-A1DC-4D39-9F54-8D3A4A866255*. The online version of this work is archived and available from the following digital repositories: PeerJ, PubMed Central and CLOCKSS.

## Systematic Palaeontology

**Table:** 

CROCODYLIFORMES Hay, 1930
MESOEUCROCODYLIA Whetstone and Whybrow, 1983
NEOSUCHIA Clark, 1988
EUSUCHIA Huxley, 1875
Genus ISISFORDIA Salisbury, Molnar, Frey and Willis, 2006

Revised diagnosis (autapomorphies marked with an ‘a’, new characters marked with an asterisk): Broad exposure of the prootic within the supratemporal foramen rostral to the rostral aperture of the posttemportal canal *(a); caudal maxillary alveolar groove *(a); maximum diameter of the caudal aperture of the cranioquadrate siphonium approximately one-third the mediolateral width of the foramen magnum, with the lateral wall of the siphonium formed exclusively by the quadrate (a); maximum mediolateral width of the secondary choanae exceeds the minimum mediolateral width of the palatines; naris with a distinctly pear-shaped outline (a); caudal dentary teeth confluent and set in a shallow alveolar groove (shared with some alligatoroids); dentary and maxillary teeth flattened labiolingually at the base of the crown, but become conical towards the apex; cervical, thoracic and cranial-most caudal vertebrae weakly procoelous at maturity (a); caudal vertebrae weakly procoelous (a); sacral vertebra II with a low caudal condyle (a); distal extremity of ulna expanded transversely with respect to the long axis of the bone (shared with *Susisuchus* spp. and *Theriosuchus pusillus*).

Remarks: An original autapomorphy listed for *Isisfordia* by Salisbury et al. (2006) was a broad exposure of the exoccipital within the supratemporal foramen. Reanalysis of the holotype by one of us (SWS) indicates that what Salisbury et al. (2006) interpreted as the exoccipital is in fact the dorsal process of the prootic. All characters listed above (except those marked with an asterisk) formed the original diagnosis of *Isisfordia* by Salisbury et al. (2006). We assign these characters to the genus *Isisfordia*, and list specific autapomorphies below.

### *Isisfordia duncani* Salisbury, Molnar, Frey and Willis, 2006

Holotype: QM F36211 (near complete skeleton, missing the rostral part of the skull).

Referred material: QM F44320 (skull).

Locality, horizon and age: Winton Formation, Albian–Turonian, Queensland.

Diagnosis: Species of *Isisfordia* with a median ridge on parietal (a); ridges on the parietal forming the medial margin of the supratemporal foramina (a); caudal maxillary tooth crown bases and alveoli ovate (a).

### *Isisfordia molnari* sp. nov.

urn:lsid:zoobank.org:act:430A065D-D1F1-459A-8984-1C43D2196FDC

Holotype: AM F125553 (braincase).

Referred material: AM F15818 (maxillary fragment).

**Figure 1:**
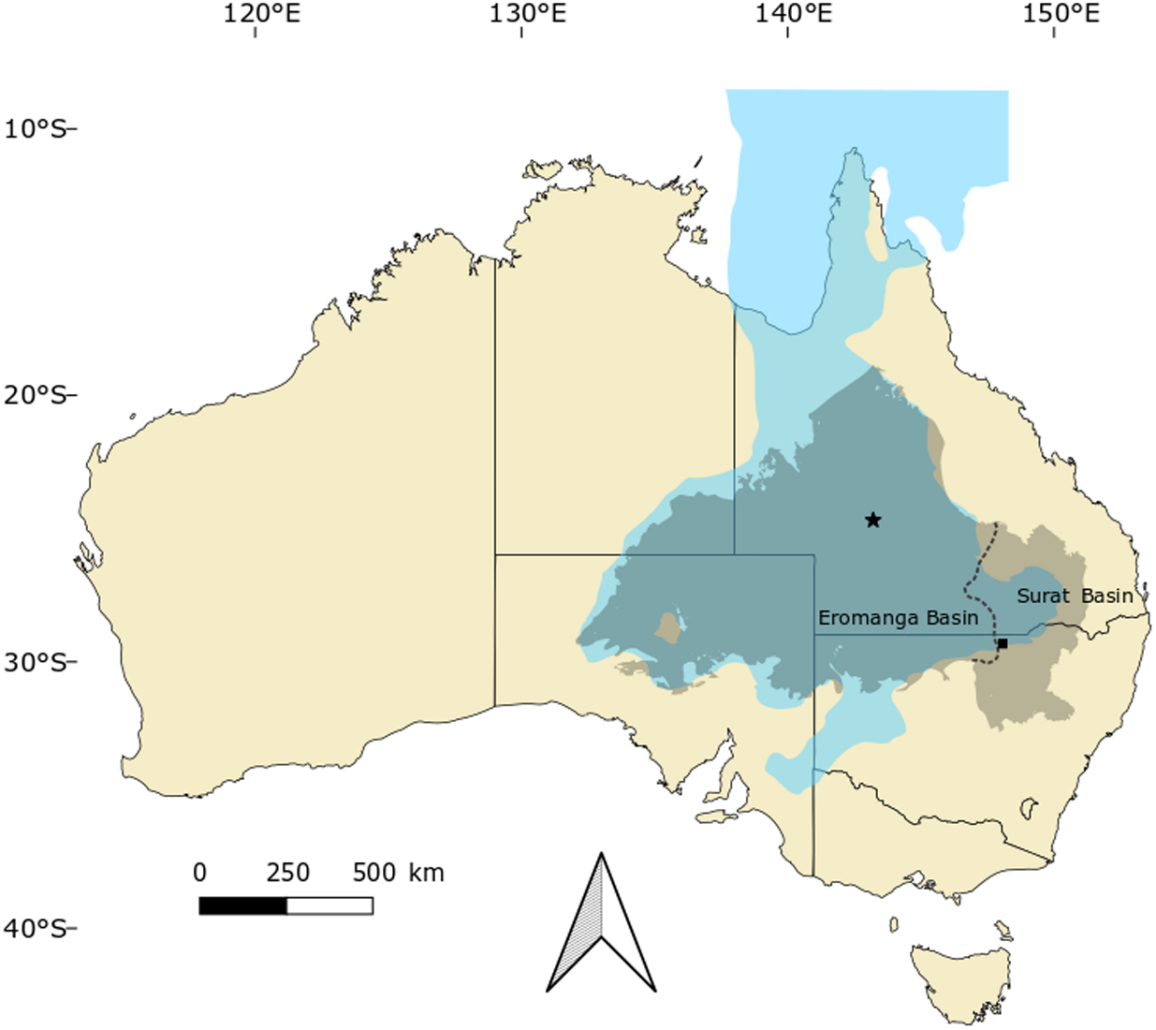
Occurrences of *Isisfordia* in the Eromanga and Surat basins of Australia. Combined, these basins form the majority of the Great Artesian Basin, which roughly approximates the outline of the Eromanga Sea (blue) during the mid-Cretaceous. Occurrences of *Isisfordia* represent: Isisford (for *I. duncani,* marked with a star), Winton Formation, uppermost Albian; Lightning Ridge (for *I. molnari* sp. nov., marked with a square), Griman Creek Formation, Cenomanian. Modified from Brougham, Smith & Bell (2017, fig. 1).

Locality, horizon and age: Both AM F125553 and AM F15818 were recovered from underground opal mines in the Lightning Ridge district, in or around the years 2000 and 1914, respectively. Precise locality data is not available; however, AM F15818 probably derives from an older field near the township, possibly the ‘Three Mile’ field. AM F125553 is likely to be from the one of the Coocoran fields, 30–40 km to the west of Lightning Ridge. Fossil-bearing rocks across the region pertain to the Wallangulla Sandstone Member of the Griman Creek Formation (part of the Surat Basin), which crops out in northern New South Wales and southeastern Queensland (Fig. 1). The Griman Creek Formation at Lightning Ridge was previously considered Albian in age (e.g., Dettman et al., 1992; Smith, 1999; Smith & Kear, 2013), but recent radiometric dating indicates a Cenomanian (96.6–100.2 Ma; Bell et al., 2019) age, slightly younger than the lower Winton Formation at Isisford (see Tucker et al., 2013). The Griman Creek Formation has produced a diverse assemblage of vertebrate fossils (see Bell et al., 2019 for a comprehensive overview) apart from crocodyliforms, including aspidorhynchids (Bell et al., 2019), lamniforms (Bell et al., 2019), dipnoans (Kemp, 1997a; Kemp, 1997b), meiolaniform and chelid testudines (Smith, 2009; Smith, 2010; Smith & Kear, 2013), leptocleidid plesiosaurs (Kear, 2006), anhanguerian pterosaurs (Brougham, Smith & Bell, 2017), ankylosaurians (Bell, Burns & Smith, 2017), small and large bodied ornithopods (Molnar & Galton, 1986; Molnar, 1996; Bell et al., 2018), titanosauriform sauropods (Molnar & Salisbury, 2005), megaraptorid theropods (Bell et al., 2015; Brougham, Smith & Bell, 2019), enantiornithes (Bell et al., 2019) and australosphenid mammals (Archer et al., 1985; Flannery et al., 1995; Clemens, Wilson & Molnar, 2003; Pian et al., 2016; T. Rich in Poropat et al., 2018).

Etymology: After palaeontologist Ralph Molnar, who has made significant contributions to Australian vertebrate palaeontology, including work on the crocodylomorphs from the Griman Creek Formation.

Diagnosis: Species of *Isisfordia* with a flat dorsal surface of the parietal (a); parietal contribution to medial margin of supratemporal fenestrae flat (does not form raised rim) (a); caudal maxillary alveoli circular and separated by interalveolar septa along entire caudal portion of the maxillary alveolar groove(a).

### AM F125553 (Braincase)

### Specimen

**Figure 2:**
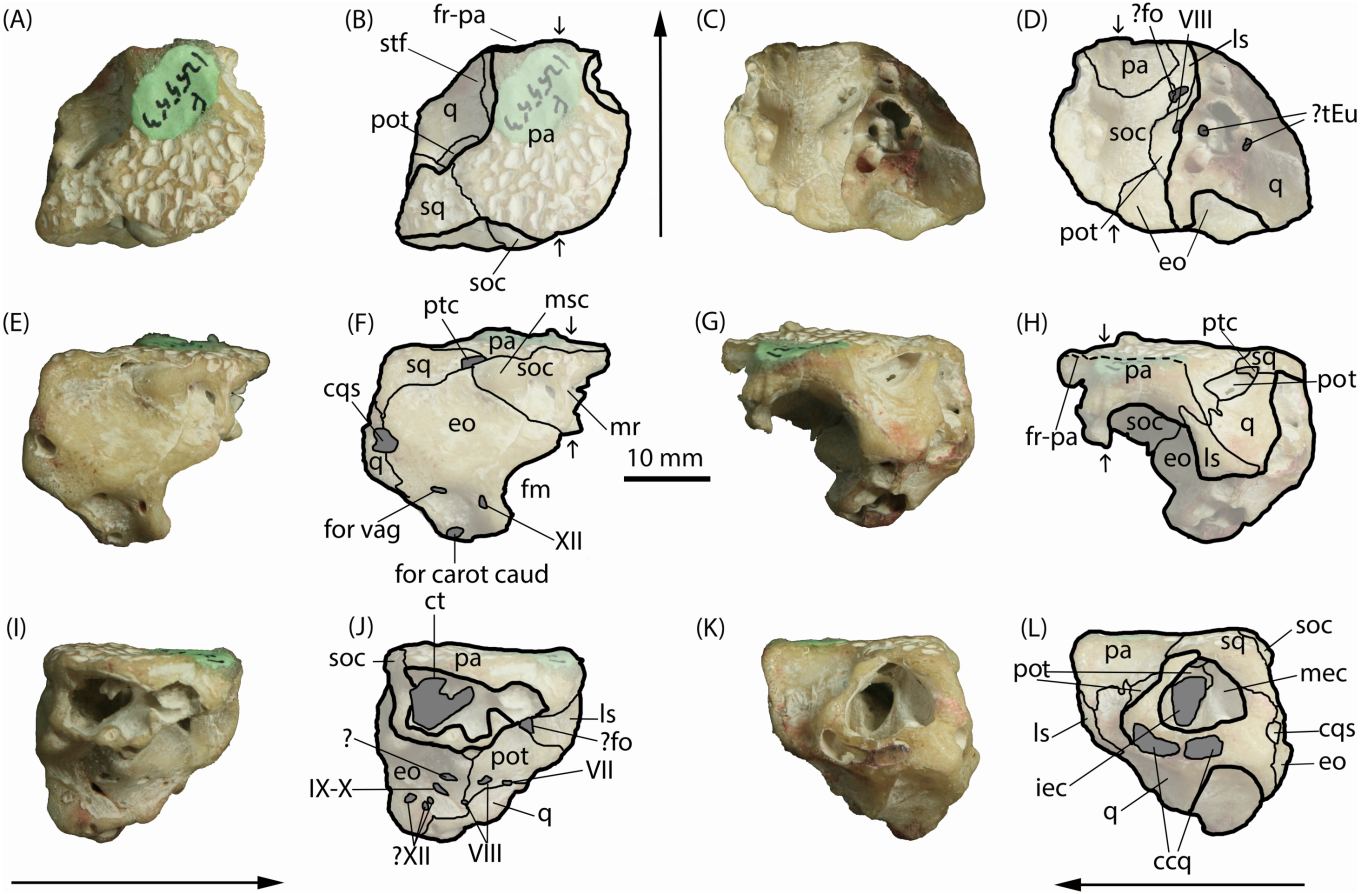
*Isisfordia molnari* sp. nov. braincase (AM F125553), photographs and interpretive line drawings. (A, B) dorsal, (C, D) ventral, (E, F) caudal, (G, H) rostral, (I, J) right lateral and (K, L) left lateral views. Small arrows in B, D, and H indicate approximate midline. Long arrows indicate rostral end in A–D and I–L. Scale bar equals 10 mm. Abbreviations: ccq, connecting canals of the quadrate; cqs, cranioquadrate siphonium; ct, transverse canal between middle ear cavities; eo, exoccipital; fm, foramen magnum; ?fo, foramen ovale; for carot caud, caudal aperture of the carotid foramen; for vag, foramen vagi; fr-pa, frontoparietal suture; iec, inner ear chamber; ls, laterosphenoid; mec, middle ear cavity; mr, median ridge for attachment of cervical muscles; msc, attachment for the *m. spinalis capitis*; pa, parietals; pot, prootic; ptc, posttemporal canal; q, quadrate; soc, supraocciptal; sq, squamosal; stf, supratemporal foramen; ?tEu, ramifications of the Eustachian tubes; IV–XII, foramina for cranial nerves IV–XII. Photo credit: Lachlan Hart.

AM F125553, a partial braincase including the fused parietals, a near complete supraoccipital and parts of the left squamosal, quadrate, exoccipital, prootic, and laterosphenoid (Fig. 2). All bones are tightly articulated and undeformed. Although opalised (preserved in honey-coloured and off-white translucent potch [non-precious opal]), AM F125553 has retained features of the bone microstructure. The specimen was purchased by the Australian Museum in the early 2000’s from opal miners Peter and Lisa Carroll.

### Description

*Parietals.* The parietals are fused, occupying roughly two-thirds of the entire specimen as it is preserved in dorsal view (Figs. 2A–2B). The rostral end appears to be broken along the frontoparietal suture, and the rostral flanges typical of crocodyliform parietals are also not preserved. The preserved area forms the medial margins of the supratemporal foramina and the caudal margin of the skull table, contacting the quadrate rostrolaterally, squamosal caudolaterally, and the supraoccipital caudally. In dorsal view, the parietals are roughly T-shaped; the caudal end of the parietals are mediolaterally broad, approximately twice as wide as the rostral end. The parietosquamosal suture is tightly interdigitating and caudomedially oriented in dorsal view. Together with the squamosal, the caudolateral extremities of the parietal form the roof of the posttemporal canal (Figs. 2F–2H), which opens rostrally into the supratemporal foramen. The prootic, which is present within the rostral aperture of the posttemporal canal, contacts the parietal at this point. Rostral to the parietal-squamosal suture, the parietals are mediolaterally constricted and the lateral margins become laterally concave (in dorsal aspect) where they form the medial margins of the supratemporal foramina. The dorsal surface of the parietals is flat and deeply pitted, typical of crocodylomorph cranial ornamentation (Iordansky, 1973). Within the supratemporal foramina, however, the surface of the parietal is smooth. Rostrally, the frontoparietal suture appears partially preserved; however, the frontal is missing and no dorsal contact between the parietals and frontal can be seen. Ventrally, the parietals form a semicircular contact with the supraoccipital (i.e., they are concave rostrally) and span the width of the endocranial cavity.

When viewed caudally, the parietals broadly contact the entire caudal width of the supraoccipital (Figs. 2E–2F). Lateral to the supraoccipital-parietal contact, the ventral edge of the parietal ascends dorsolaterally (in caudal view) to form a portion of the medial border of the caudal aperture of the posttemporal canal and overlapping the reciprocal medial process of the squamosal. In right lateral view (Figs. 2I–2J), the parietal contacts the supraoccipital caudally, and the laterosphenoid rostroventrally.

*Squamosal.* The left squamosal, which forms the caudolateral corner of the skull roof, is broken laterally, exposing the prootic and the large middle ear cavity, which mostly resides within the quadrate (Figs. 2K–2L). Similar to the parietals, the squamosal is ornamented dorsally by an irregular series of pits. The short medial process of the squamosal partly roofs the posttemporal canal, contacting the parietal along an interdigitating, caudomedially-oriented suture in dorsal view (Figs. 2A–2B). Lateral to this canal, the rostral margin of the medial process of the squamosal is V-shaped (i.e., concave rostrally) in dorsal view, forming a small portion of the caudal boundary of the supratemporal foramen. The squamosal contributes a short descending process that forms part of the lateral wall of the posttemporal canal in rostral view, where it contacts the prootic ventromedially and the quadrate rostrally.

In caudal view, the squamosal is sub-triangular as preserved (Figs. 2E–2F), forming the roof of the posttemporal canal at its medial corner, where it underlaps the caudolateral edge of the parietal. The ventral margin (in caudal view) is sinuous, forming a broad contact with the exoccipital.

*Quadrate.* The left quadrate, as preserved, is a large, complex bone, which forms much of the lateral wall of the braincase. It contacts (from rostral to caudal) the parietals, prootic, exoccipital and squamosal dorsally, the laterosphenoid rostrally, and the prootic and exoccipital medially. The caudal surface of the quadrate is worn, obliterating the articular condyles.

The quadrate makes up most of the preserved lateral and ventrolateral faces of the braincase (Figs. 2C–2D; 2K–2L). These surfaces of the quadrate are extensively damaged and worn, so distinct external morphologies and sutures are not clear. However, this damage has exposed parts of the middle ear cavity. In ventral view, six distinct openings are visible. The caudal-most opening is ovoid, oriented rostrolaterally, and formed by both the quadrate and exoccipital. Immediately rostral to this opening is a second small, circular opening that appears to be fully enclosed by the quadrate; this, and another small more-medially situated opening, could potentially be ramifications of the Eustachian tubes. The rostral-most opening is the largest of the five and is situated rostrolaterally relative to the caudal two openings. It is somewhat reniform externally and extends dorsally into the skull to form part of the inner ear.

Caudal to the exposed chambers of the middle and inner ear, in lateral view, the quadrate expands dorsally to contact the squamosal dorsally and the exoccipital caudally and medially (Figs. 2E–2F; 2K–2L). The large cranioquadrate siphonium is formed by the quadrate laterally and exoccipital medially. Ventral to the middle and inner ear chambers, is a rostrocaudally elongate opening exposed on the abraded lateral surface of the quadrate (Figs. 2K–2L). Within this artificial exposure are two openings, interpreted as the connecting canals of the quadrate (Iordansky, 1973).

The dorsal surface of the quadrate is rostroventrally sloping and mediolaterally concave, forming the floor within the supratemporal foramen. Caudodorsally, the quadrate contacts the prootic along a caudally-concave suture rostral to the rostral aperture for the posttemporal canal. On either side of this opening, the quadrate rises to meet the squamosal laterally and the parietal medially, forming the lateral and medial walls of the rostral aperture for the posttemporal canal. Rostrally, the quadrate contacts the laterosphenoid along a caudally-concave suture; however, the specimen is broken and abraded along this suture, obscuring most of its details. The medial surface of the quadrate, exposed in right lateral view (Figs. 2I–2J), is bordered dorsally by the exoccipital and the prootic more rostrally.

*Exoccipital.* When viewed caudally, the dorsal margin of the exoccipital is broadly triangular, contacting the squamosal dorsolaterally and supraoccipital dorsomedially. Ventral to the supraoccipital contact, the medial edge of the exoccipital is medially concave, forming the lateral border of the foramen magnum, which occupies roughly half of the preserved height of the exoccipital. Although the right exoccipital is not preserved, a ventral extension of the supraoccipital appears to have prevented the left and right exoccipitals from contacting one another along the dorsal margin of the foramen magnum (Figs. 2E–2F). However, without the right exoccipital being present this cannot be diagnosed with certainty. Lateral to the foramen magnum, the exoccipital is pierced by three foramina of equal size, which lie within a well-defined fossa on the basioccipital process of the exoccipital. The medial-most foramen corresponds to the opening for c.n. XII. The caudal aperture of the carotid foramen lies ventrolateral to the opening for c.n. XII on the ventral-most edge of the exoccipital, and the foramen vagi lies dorsal the caudal aperture of the carotid foramen. The lateral margin of the exoccipital, below the level of the squamosal contact, is convex (in caudal view), interrupted at about its mid-height by the opening for the cranioquadrate siphonium. The exoccipital forms the medial wall of that opening. The ventral end of the exoccipital (basioccipital process) is broken and worn, therefore its relationship with the basioccipital is unknown.

In ventral view, the medial edge of the exoccipital contacts the supraoccipital and prootic creating a sinuous rostrocaudal suture. The contacts between the exoccipital and quadrate are largely obscured by fusion.

The medial exposure of the exoccipital, within the endocranial cavity, reveals several foramina (Figs 2I–2J). The caudal-most of these is a trio of small openings potentially corresponding to c.n. XII. Rostrodorsal to the rostral-most of these openings is the elliptical foramen for c.n. IX-X, which sits within a shallow rostroventrally-caudodorsally oriented groove. Dorsal to this opening is another cavity of unknown origin (Iordansky, 1973) and could be a preservational artefact. Rostrovental to this last opening is the first foramen of c.n. VIII, which resides on the exocciptal-prootic suture where it makes contact ventrally with the quadrate.

*Supraoccipital.* The supraoccipital is lacking most of its right side but, when complete, would have formed an inverted triangle (flat dorsally) in caudal view (Figs. 2E–2F). It is divided caudally by a median ridge that would have provided attachment for the cervical muscles (Iordansky, 1973). This ridge terminates ventrally in a slightly expanded, teardrop-shaped process that appears to have contributed to the dorsal-most margin of the foramen magnum (Figs. 2C–2F). This process is also visible in ventral view, bound by the exoccipital laterally (Figs. 2C–2D).

Caudally, on either side of this median ridge, is a deep, hemispherical fossa. Lateral to this fossa, the dorsolateral corners of the supraoccipital are distinctly bulbous, and delimit the posttemporal canal medially. This bulbous process would have served as the attachment point for the m. spinalis capitis (Iordansky, 1973).

The most salient feature of the supraocciptal is a large exposure of the transverse canal, connecting the middle ear cavities, visible in right lateral view. This is an elliptical cavity (in right lateral aspect), punctuated by a small bony process that descends from the ventral wall of the parietal, situated approximately a third of the way along the depth of the cavity.

The ventral surface of the supraoccipital is mediolaterally concave, forming part of the roof of the endocranial cavity (Figs. 2C–2D). Descending processes of the parietals contact the exoccipitals, together forming the lateral walls of the endocranial cavity. A medial expansion of the exoccipital and a caudal expansion of the parietals convey an hour-glass shape to the supraoccipital in ventral aspect. These expansions of the exoccipital correspond to the widening of the large middle ear cavity, which is partly visible within the broken descending processes of the exoccipital and quadrate on the right side (Figs. 2C–2D).

*Laterosphenoid.* A portion of the caudal margin of the laterosphenoid is preserved. Externally, the laterosphenoid contacts the quadrate caudally and the parietal dorsally (Figs. 2G–2H). Medially, within the endocranial cavity, the preserved portion of the laterosphenoid is roughly triangular, bound by the parietal dorsally and the prootic ventrally and caudally (Figs. 2I–2J). An opening, which potentially pertains to the *foramen ovale,* is situated at the dorsal end of the laterosphenoid-prootic suture. In lateral aspect, the laterosphenoid is bound along its rostrodorsally-inclined caudal margin by the parietal dorsally, and, more extensively, by the quadrate ventrally (Figs. 2K–2L).

**Figure 3:**
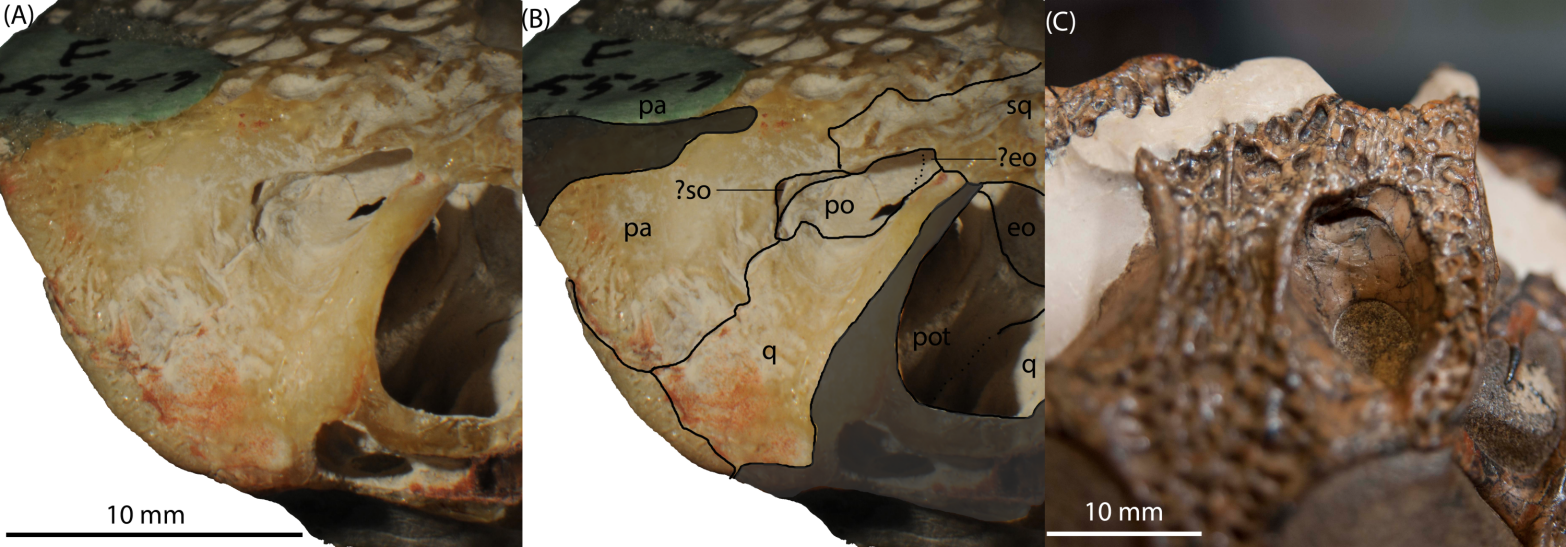
*Isisfordia molnari* sp. nov. braincase (AM F125553) in left lateral oblique view, showing broad exposure of the prootic within the supratemporal foramen. (A) unmodified photograph, (B) interpreted sutures. Rostral is to the left. (C) Rostrodorsal view of *I. duncani*, showing the same feature. Scale bar equals 10 mm. Shaded area indicates broken/abraded surfaces. Abbreviations: eo, exoccipital; ls, laterosphenoid; pa, parietal; pot, prootic; q, quadrate; so, supraoccipital; sq, squamosal. Photo credit: A, B: Phil Bell; C: Steve Salisbury.

*Prootic.* The prootic is exposed in left lateral view (Figs. 2K–2L) as a rostrocaudally oriented bar within the middle ear cavity. It forms the dorsal boundary of the inner ear cavity, with the dorsal process of the prootic emerging to form part of the ventral surface of the supratemporal foramen as a tongue-shaped process (Figs. 2A–2B; 3A–3B), and separating the exoccipital from the quadrate within the foramen (Figs. 2G–2H). In right lateral view (Figs. 2I–2J), the prootic is roughly triangular in shape, contacting the laterosphenoid rostrodorsally, the quadrate ventrally and the exoccipital caudally. The opening for c.n. VII resides on the prootic-quadrate suture. The rostral-most opening for c.n. VIII is formed entirely by the prootic and located caudal to the opening for c.n. VII. In ventral view (Figs. 2C–2D), the prootic is semicircular (convex medially) forming a bulge on the lateral wall of the endocranial cavity that houses part of the labyrinth of the middle ear.

### AM F15818 (Maxillary fragment)

#### Specimen

**Figure 4:**
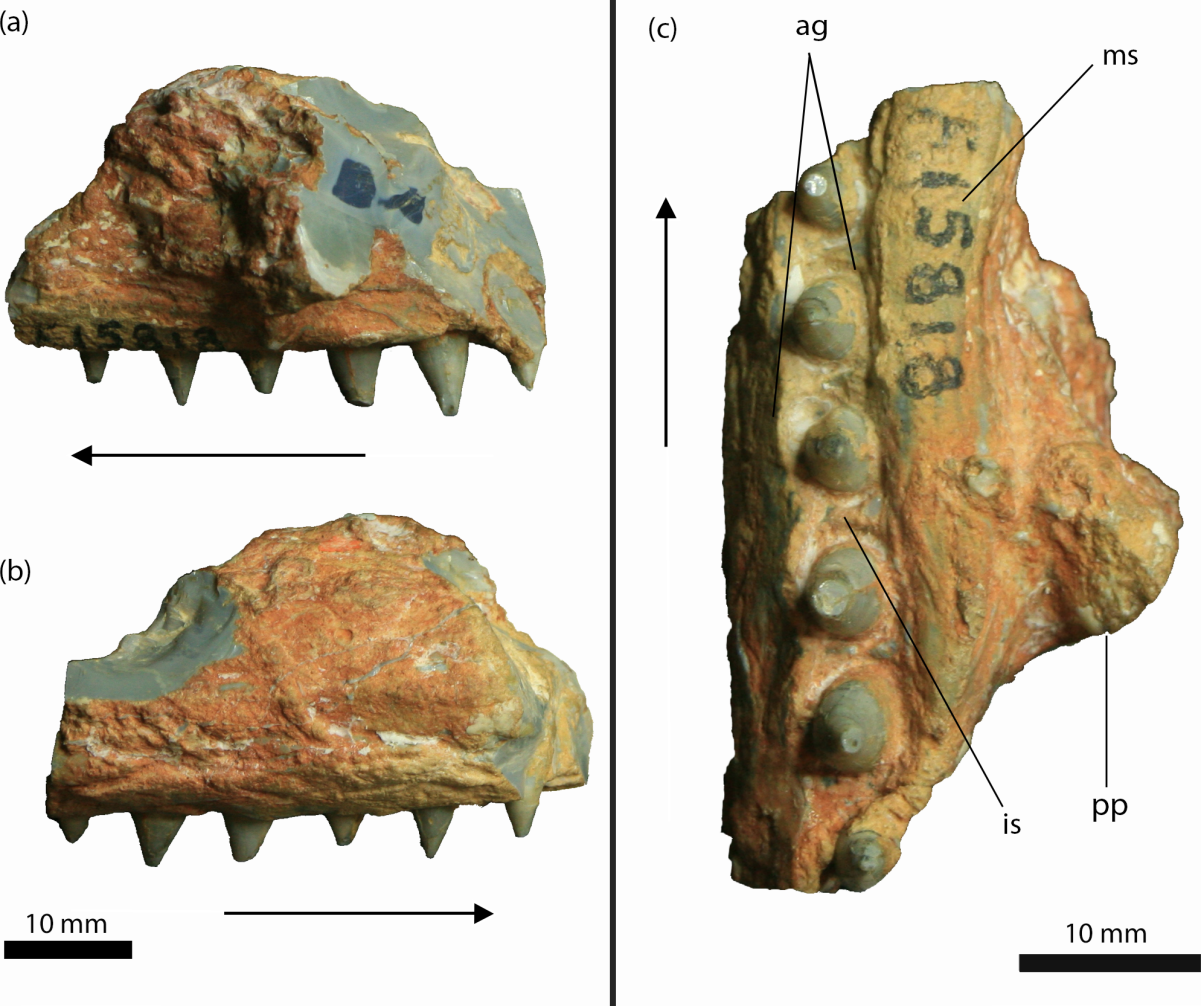
*Isisfordia molnari* sp. nov. maxillary fragment (AM F15818) from the Griman Creek Formation. (A) medial, (B) lateral, (C) palatal views. Arrows indicate rostral end. Scale bars equal 10 mm. Abbreviations: ag, alveolar groove; is, interalveolar septa; ms, medial ‘shelf’; pp, palatal process. Photo credit: Lachlan Hart.

AM F15818, a maxillary fragment bearing six teeth, previously designated as the holotype of ‘*Crocodylus (Bottosaurus) selaslophensis*’ (Fig. 4) and donated to the Australian Museum in 1914 by Colonel R. E. Roth (Etheridge, 1917; Smith, 1999). The entire specimen is preserved as a pseudomorph in common blue-black opal (SiO_2_*n* H_2_O), which has obliterated the internal features of the bone.

#### Description

**Figure 5:**
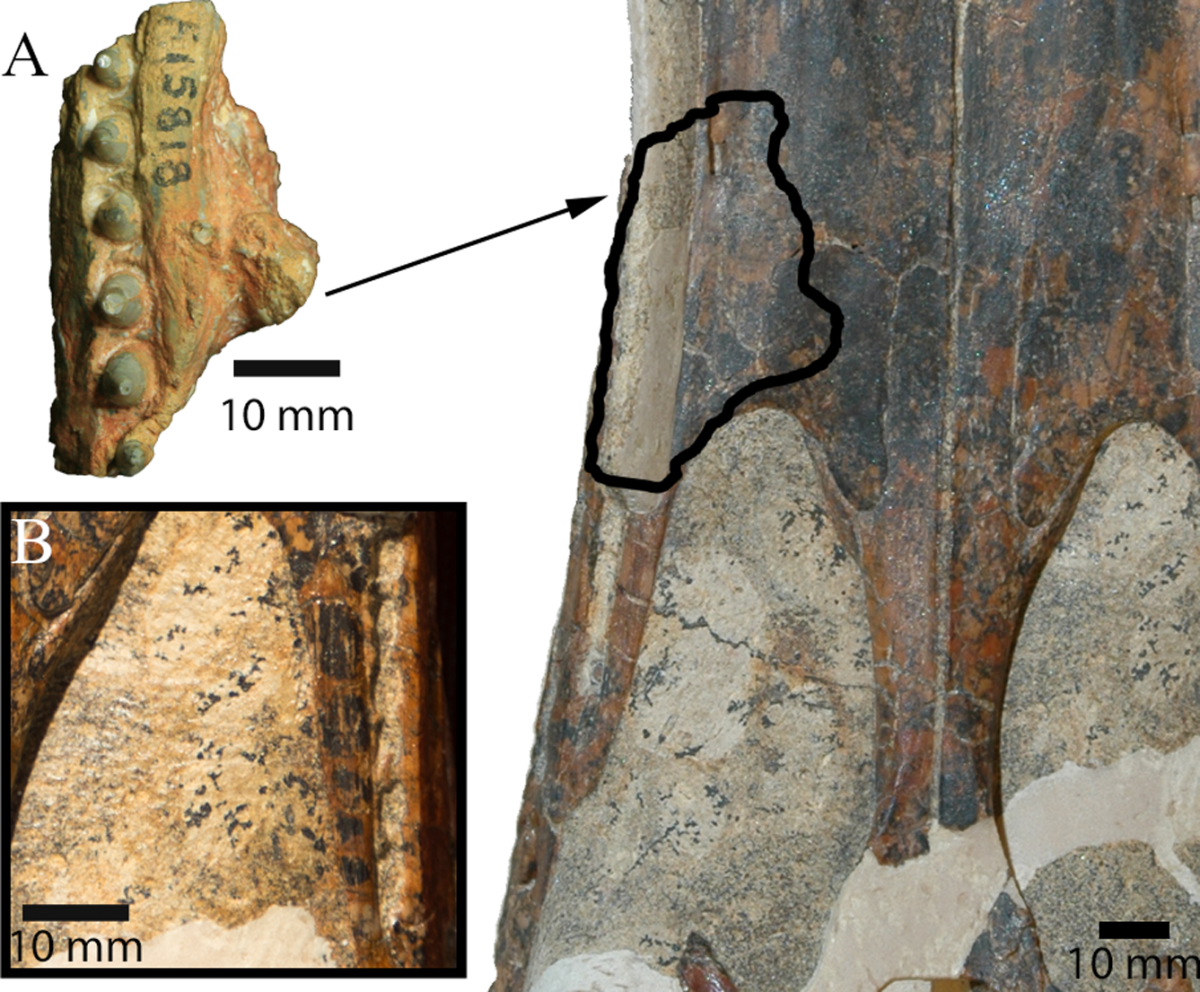
Comparison between *I. duncani* and *I. molnari*. (A) Comparison between AM F15818 (left) and QM F44320 (paratype of *I. duncani*; right). Outline on QM F44320 shows hypothesised location of AM F15818. (B) Detail of left caudal maxillary alveolar groove on QM F44320 in palatal view. Scale bars equal 10 mm. Photo credits: QM F44320 photographs by Steven Salisbury, AM F15818 photograph by Lachlan Hart.

AM F15818 is an incomplete section (maximum length = 39 mm; maximum mediolateral width = 25 mm; dorsoventral depth = 25 mm, not including the teeth) of the right maxilla (Figs. 4 and 5). The lateral surface of the maxilla is dorsoventrally convex and rugose, ornamented by deep longitudinal grooves. Further details of the ornamentation or possible foraminae are obscured by a thin layer of brown-orange matrix. On either side of the dentition, the medial and lateral walls of the maxilla are thickened, forming mediolaterally convex “shelves” (Molnar, 1980, pg. 66). The lateral and medial shelves are approximately as wide as the alveolar groove (see below), forming a broad ventral surface. A small broken section of bone, which we interpret as the palatal process, is preserved medially, extending along the rostral two-thirds of the fragment and up to ∼5 mm medially from the tooth-bearing segment. The caudal margin of this process forms an acute angle that, in our interpretation, would have circumscribed the rostral boundary of the right suborbital fenestra. As described by Etheridge (1917) and revised by Molnar (1980), AM F15818 bears six teeth set within an alveolar groove. Individual teeth are separated by prominent interalveolar septa along the entire preserved tooth row (Fig. 4). The crowns are subconical (crown height = ∼5 mm) and lingually curved. There are faint non-denticulate mesial and distal carinae on the crowns but no other surface ornamentation is present. The crown-root junction is unclear, as the crowns are only slightly labiolingually flattened at their base and there is no definition of enamel or dentine preserved on the tooth (an artefact of the opalised preservation). The root is exposed on the rostral-most tooth. It is lingually curved and is approximately three times the height of the corresponding crown.

## Discussion

Salisbury et al. (2006) diagnosed *Isisfordia duncani,* in part, based on the possession of two unique features of the braincase: (1) a broadly exposed exoccipital within the supratemporal foramen, rostral to the rostral aperture of the posttemporal canal, and; (2) maximum diameter of the caudal aperture of the cranioquadrate siphonium approximately one-third the mediolateral width of the foramen magnum, with the lateral wall of the siphonium formed exclusively by the quadrate. Regarding the first feature, our examination of the holotype of *I. duncani* re-identifies what Salisbury et al. (2006) interpreted as the exoccipital, as the dorsal process of the prootic (Fig. 3C). A similar broad exposure of the prootic within the supratemoral foramen is apparent on AM F125553, emerging rostral to the rostral aperture of the posttemporal canal and bound by the quadrate, squamosal and parietal (Figs. 3A and 3B). In the closely-related taxon *Susisuchus jaguaribensis* (Fortier & Schultz, 2008), the prootic does not extend into the supratemporal foramen. This is also the case in the basal eusuchians *Hylaeochampsa vectiana* (Clark & Norell, 1992) and *Iharkutosuchus makadii* (Osi, Clark & Weishampel, 2007).

Regarding the second feature, the foramen magnum is incomplete in AM F125553, but its width can be estimated based on the well-preserved left side of the braincase. The estimated width of the foramen magnum (∼11 mm), equates to approximately three times the maximum width of the caudal aperture of the cranioquadrate siphonium (∼3.5 mm; see Figs. 2E–2F). Furthermore, in *Susisuchus* spp. and crocodylians, the lateral wall of the cranioquadrate siphonium is formed by the exoccipital, whereas in *I. duncani* and AM F125553, the cranioquadrate siphonium is formed by the quadrate, laterally, and the exoccipital, medially (Figs. 2E–2F; 2K–2L). The presence of these autapomorphies allow the referral of AM F125553 to *Isisfordia*; however, the latter feature can only be tentatively identified in AM F125553, as the lateral surface of the quadrate—which contributes to the cranioquadrate siphonium—is worn, so the exact measurements of this area cannot be ascertained with confidence.

An important point of difference between *I. duncani* and AM F125553 is the dorsal morphology of the parietals. In *I. duncani,* the parietal has a defined median ridge, and lateral ridges forming the medial margin of the supratemporal foraminae, which are visible on both the holotype and paratype specimens (figs. 2 and 4, Salisbury et al., 2006). Conversely, in AM F125553, these ridges are absent and the dorsal surface of the parietals is flat (Fig. 2A). As AM F125553 is significantly larger than the largest known specimens of *I. duncani,* we discount this as an ontogenetic feature, and recognise this trait as distinct between species.

Another feature which warrants further discussion is the morphology of the supraoccipital of AM F125553. As described above, it appears to contribute to the dorsal margin of the foramen magnum (Figs. 2E–2F). This is not seen in *I. duncani*, where the dorsal margin of the foramen magnum is composed entirely of the exoccipitals (Salisbury et al., 2006), and as such, represents an additional possible autapomorphy of *I. molnari*. However, due to the incomplete nature of AM F125553, this cannot be determined with certainty, and thus we refrain from including this feature in the species diagnosis.

As AM F125553 displays characteristics diagnostic of *Isisfordia*, it is practical that it be classified as such. The unique presentation of the parietals, and possibly the supraoccipital, are due cause to separate AM F125553 from *I. duncani,* and permit its recognition as a distinct species.

Both Etheridge (1917) and Molnar (1980) interpreted AM F15818 as a dentary fragment, although they disagreed as to which part of the dentary it belonged (Etheridge, 1917 believed it was from near the symphyseal region, but Molnar, 1980 disagreed). The interpretation of AM F15818 as a maxillary piece was previously considered, but not entertained in Molnar (1980) because of the dorsoventral depth of the presumed palatal process (R Molnar, pers. comm., 2019). Smith (1999) was the first to propose that the specimen was a maxillary fragment, which is the interpretation that we follow here. In fact, several of the features described by Molnar (1980) support this interpretation. Molnar (1980, pg. 66) described a “medial shelf” that extended along the tooth row of AM F15818. Here, we have interpreted this feature as a medial thickening of the maxilla where it forms the lateral margin of the suborbital fenestra, a feature not seen in *I. duncani*, and unique to AM F15818. However, as this feature may be an artefact of preservation rather than a reflection of true morphology, it has not been included in the specific diagnosis for *I. molnari*. Molnar (1980, pg. 66) also described a “prominent mass” of opal on the medial surface of AM F15818, which he interpreted as either a displaced bone fragment or an opalised mass of matrix, and not a natural projection from the fossil. However, this assertion appears to be based on Etheridge’s (1917) initial assumption that AM F15818 was a dentary fragment. There is no evidence of separation between this “mass” and the main part of the jaw that might have indicated that these represent separate entities (either bone or opal). When viewed as a maxillary fragment, however, this projection likely represents a caudal section of the medial palatal process, which formed the rostral boundary of the suborbital fenestra. The configuration of this process also indicates that AM F15818 derives from the right side of the skull (Fig. 5). Alternatively, AM F15818 might have derived from further caudally on the maxilla, with the “mass” representing a portion of the ectopterygoid, circumscribing part of the caudolateral margin (as opposed to the rostral margin) of the left suborbital fenestra. However, this configuration would have extended the tooth row further caudally than is present in *I. duncani* and probably *Susisuchus* (Leite & Fortier, 2018).

Perhaps the most salient feature of AM F15818 is the alveolar groove, which Molnar (1980) even suggested threw the crocodyliform identification of AM F15818 into doubt. An alveolar groove is an uncommon feature in crocodyliforms; however, it is present in *Isisfordia duncani*, alligatoroids and *Hylaeochampsa vectiana* (Salisbury et al., 2006). In *I. duncani,* the caudal-most dentary and maxillary teeth sit within such a groove. Although an alveolar groove is present in *H. vectiana*, whether or not the caudal teeth also resided in a groove is unknown as this section of the skull has not been recovered (Clark & Norell, 1992). In other hylaeochampsids such *Iharkutosuchus makadii*, the greatly enlarged size of the caudal-most maxillary alveoli relative to the more rostral ones (see Osi, Clark & Weishampel, 2007) renders meaningful comparisons with AM F15818 and *I. duncani* difficult. Regardless, the combination of an alveolar groove and the preserved rostral margin of the suborbital fenestra indicates that AM F15818 is also from the caudal-most part of the tooth row.

The tooth morphology of AM F15818 differs from that of *I. duncani.* As reported by Salisbury et al. (2006), the teeth of *I. duncani* can be differentiated from those of alligatoroids in being labiolingually flattened at the crown base with a convex (in mesiodistal view) labial surface and concave lingual surface (unlike the bulbous teeth seen in the alveolar groove of alligatoroids (fig. 4, Salisbury et al., 2006). These same features differentiate AM F15818 from alligatoroids. Indeed, while discussing the dental features of *I. duncani,* Salisbury et al. (2006) commented on its similarity to AM F15818 (referred to as a “putative eusuchian”; p. 2442). The morphology of the caudal-most teeth in *Hylaeochampsa* is unknown (Clark & Norell, 1992; Salisbury et al., 2006), but in related hylaeochampsids such as *Iharkutosuchus makadii,* the caudal maxillary teeth are broad, multicusped and molariform (Osi, Clark & Weishampel, 2007). The teeth of AM F15818 differ from those of *I. duncani* as they are not as strongly labiolingually flattened and are more rounded at the crown base (fig. 4, Salisbury et al., 2006). The corresponding alveoli are also rounded, as opposed to the ovate alveoli seen in *I. duncani*.

Interdental septa are present between all teeth in the alveolar groove of AM F15818, but are not present between all teeth in the alveolar groove in *I. duncani* (QM F44320), instead being present at the rostral end of the alveolar groove and becoming discontinuous (i.e., incompletely separating adjacent alveoli) caudally (Fig. 5B; see also fig. 4, Salisbury et al., 2006). This feature is ontogenetically variable in modern alligatoroids (C Brochu, pers. comm., 2019), and in those cases may not be taxonomically informative. However, the paratype skull of *I. duncani* comes from an individual that was larger than the holotype, the latter of which represents a mature individual based on fusion of the neurocentral sutures on all cervical vertebrae (except the axis) (Salisbury et al., 2006). On this basis, the paratype skull (on which we based many of our comparisons) also is likely to have derived from a full size, mature individual. The reduced interalveolar septa in *I. duncani* are therefore a feature of a mature individual. The maxilla of *I. molnari* represents a much larger (and therefore presumably mature) individual than the paratype of *I. duncani*. The presence of the continuous interalveolar septa in *I. molnari* therefore cannot be dismissed as ontogenetic variation and are a key difference between the two species.

The combination of the unusual alveolar groove, and the labiolingually compressed, lingually curved crowns of AM F15818 allow its referral to *Isisfordia*. The presence of the “medial shelf” (thickening of the medial alveolar wall), and differences in the crown morphology, alveolar shape, and the unique arrangement of the caudal maxillary alveolar septa between AM F15818 and *I. duncani* are due cause to assign the former to a separate species. AM F15818 can confidently be removed from *Crocodylus* as it is not morphologically or temporally congruent with any known *Crocodylus* species; as previously mentioned, an alveolar groove has only been seen in *Isisfordia duncani*, alligatoroids and *Hylaeochampsa vectiana* (Salisbury et al., 2006). Temporally, the fossil record of the *Crocodylus* genus extends only back to the Miocene (Brochu, 2000), some 80 million years younger than the age given for the Griman Creek Formation (Bell et al., 2019) from which AM F15818 and AM F125553 derive. Likewise, as already observed by Molnar (1980), *Bottosaurus* is a genus of alligatoroid known only from North America.

Following the reassignment of AM F15818 to *Isisfordia molnari* sp. nov., a re-evaluation of other crocodyliform material from the Griman Creek Formation, such as those described by Molnar (1980) and Molnar & Willis (2001), is necessary but beyond the scope of this paper. Central to this reasoning is the fact that the original specimens described by Molnar & Willis (2001) are missing and could not be relocated at the time of writing. Molnar & Willis (2001) described these remains as representative of a putative ‘ziphodont’ crocodyliform, and, as a result, removed a number of additional isolated and non-overlapping remains previously referred to *‘C. selaslophensis’* by Molnar (1980) from the latter taxon. We find that the identification by Molnar & Willis (2001) of a small, broad-snouted mesoeucrocodylian with laterally compressed teeth is congruent with *Isisfordia.* In light of this, it is quite plausible that all previously described crocodyliform material from Lightning Ridge belongs to a single taxon (Molnar, 1980), which we here identify as *I. molnari.* A detailed reappraisal of other cranial and postcranial Lighting Ridge crocodyliform material is being undertaken by the first author and will be presented elsewhere.

The occurrence of a new species of *Isisfordia* in the Griman Creek Formation is significant in several respects. These findings increase the geographic range of the genus *Isisfordia*: Isisford and Lightning Ridge are separated by roughly 800 km (Fig. 1), although they were roughly coeval (Isisford is best described at 100.5–102.2 Ma (Syme et al., 2016); Lightning Ridge is currently considered 96.6–100.2 Ma (Bell et al., 2019). Geographic ranges of this size can also be observed in extant crocodilians (see Kushlan & Mazzotti, 1989; Letnic & Connors, 2006; Grigg & Kirshner, 2015).

During the middle Cretaceous, Isisford and Lightning Ridge were located at high latitudes; 54°S and 58°S, respectively (palaeolatitudes obtained using GPlates 2.0; utilising data sets after (Matthews et al., 2016; Amante & Eakins, 2009). Mean annual average temperature estimates based on floral signatures and oxygen isotopes for the Griman Creek Formation are 12–16 °C; (Dettman et al., 1992). This is indirectly supported by the diverse record of meso-reptiles (specifically, non-marine turtles) from the same formation (Smith, 2009; Smith, 2010; Smith & Kear, 2013). A similar temperature range of 10–18 °C has been determined for the Winton Formation at Isisford (Syme & Salisbury, 2018). These temperatures are congruent with the thermal tolerance of extant crocodilians (∼14 °C mean annual temperature), suggesting that *I. duncani* and *I. molnari* both possessed similar thermal tolerances to modern crocodilians.

**Figure 6:**
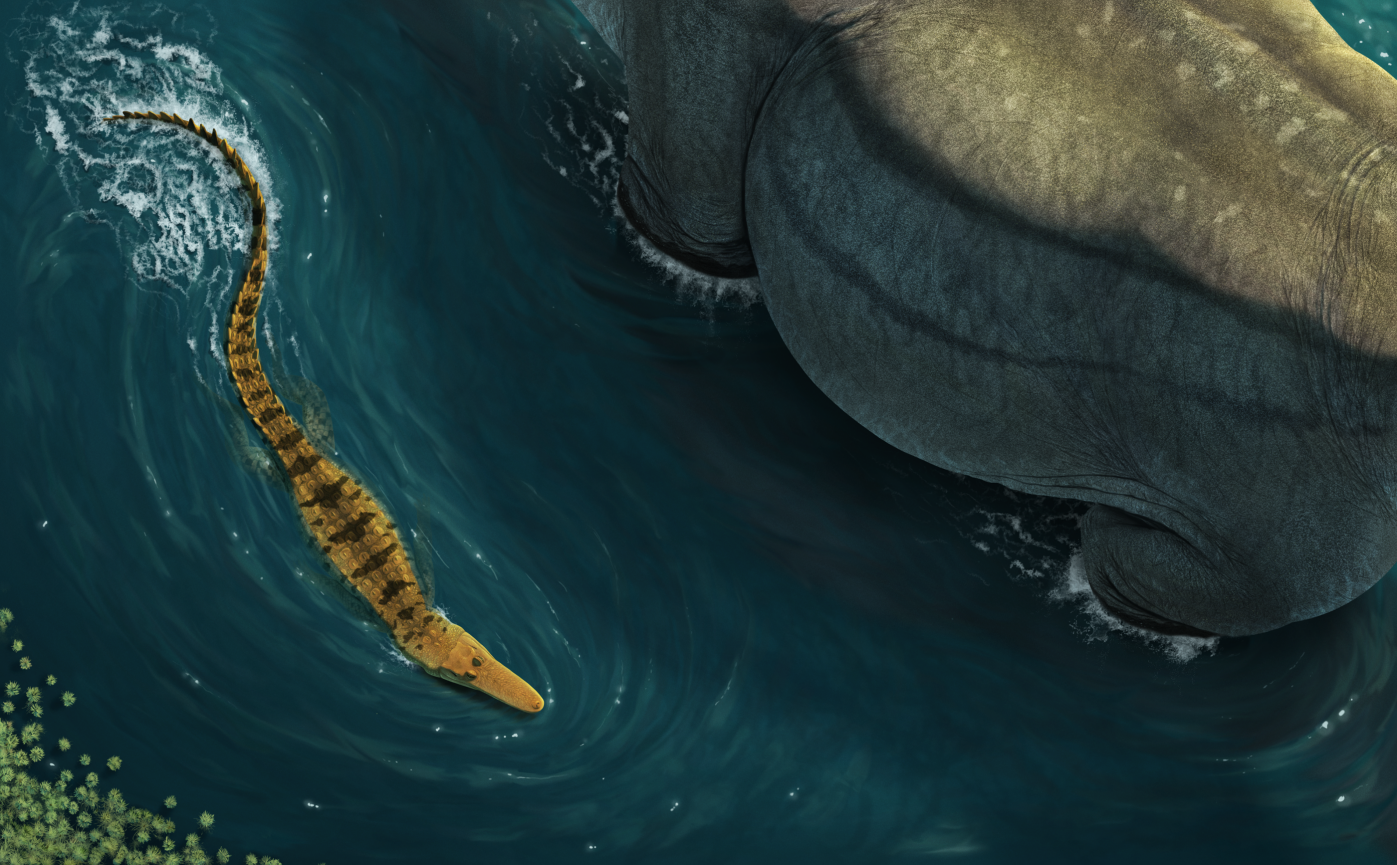
Reconstruction of *Isisfordia molnari* sp. nov. In life mode, swimming next to a wading sauropod. Reconstruction by José Vitor Silva.

*Isisfordia* currently appears to be confined to the floodplains and near-coastal terrestrial environments on the eastern margin of the Eromanga Sea (Fig. 6). The faunal assemblage at Isisford is considered to represent a marginal marine to very distal continental (transitional) system (Tucker et al., 2017), deposited under fluctuating energy levels in a brackish water setting (Syme et al., 2016). The Griman Creek Formation is interpreted as a lacustrine to estuarine coastal floodplain (Dettman et al., 1992; Bell et al., 2019), deposited under cold conditions in an oxygen-deprived fluvial-deltaic environment (Rey, 2013). Both these palaeoenvironments represent coastal lowland settings that drained into the adjacent epeiric Eromanga Sea, which is comparable to the environmental preferences of extant crocodilians (see Grigg & Kirshner, 2015; Ch. 10).

*Isisfordia duncani* is significant as it traditionally represents the basal-most eusuchian (Salisbury et al., 2006). Different analyses have recovered *I. duncani* as an advanced neosuchian (Turner & Pritchard, 2015), or as the sister taxon of *Susisuchus* within Eusuchia (Leite & Fortier, 2018). Regardless of the phylogenetic position of *I. duncani,* the taxon represents an evolutionary milestone in the crocodyliform lineage, possessing features considered transitional between ‘advanced’ neosuchians and eusuchians (see Leite & Fortier, 2018). The identification of a second species of *Isisfordia* from the roughly coeval Griman Creek Formation demonstrates that the genus was well-established in eastern Gondwana during the mid-Cretaceous. *Isisfordia* is the first Australian Mesozoic archosaur to be known from multiple distinct species, further underscoring the paucity of Australia’s Mesozoic terrestrial vertebrate fossil record. The new occurrence clarifies systematic aspects of the enigmatic Griman Creek Formation crocodyliforms and provides evidence that basal eusuchians had become established and widespread in eastern Australia by the mid-Cretaceous.

## Conclusions

*Isisfordia molnari* sp. nov., from the Griman Creek Formation, is described based on a partial braincase (AM F125553). This specimen is assigned to *Isisfordia* based on the presence of one unambiguous (and newly-defined) autapomorphy: a broadly exposed prootic within the supratemporal foramen, rostral to the rostral aperture of the posttemporal canal. Differences in the morphology of the parietals and the apparent inclusion of the supraoccipital in the dorsal margin of the foramen magnum of AM F125553 warrant the exclusion of AM F125553 from *I. duncani*. A maxillary fragment, AM F15818 (formerly the holotype of *‘Crocodylus (Bottosaurus) selaslophensis’*), is also referred to this new species. This is based on unique features of the dentition and the presence of an alveolar groove, shared only with *Isisfordia duncani* from the Lower Cretaceous portion of the Winton Formation. We justify specific separation based on differences in alveolar and tooth crown base morphology, and the presence of interalveolar septa in the caudal part of the maxillary toothrow and a thickening of the medial alveolar wall of AM F15818.

*Isisfordia* represents the first multispecific Australian Mesozoic archosaur, underscoring Australia’s poor Mesozoic terrestrial vertebrate fossil record. In light of these findings, a reappraisal of all previously-described crocodyliform material from the Griman Creek Formation is now pertinent, although future work will continue to be hampered by the isolated and often fragmentary specimens that are the result of the unique mining setting in which these fossils are found. Nevertheless, we agree with Molnar (1980) that Griman Creek Formation crocodyliform material likely derives from a single taxon (identified here as *Isisfordia molnari*), providing a working hypothesis on which to base future work.

## Supplemental Information

10.7717/peerj.7166/supp-1 Supplemental Information 1 Measurement data Abbreviations: WFM: Width of Foramen Magnum WCACS: Width of caudal aperture of the cranioquadrate siphonium ML: Maximum length MMW: Maximum mediolateral width DD: Dorsoventral depth.

## Additional Information and Declarations

### Data Availability

The following information was supplied regarding data availability: Holotype and referred material of Isisfordia duncani is stored at the Queensland Museum (QM). Accession numbers: QM F36211 (holotype); QM F44320 (referred). Holotype and referred material of Isisfordia molnari is stored at the Australian Museum (AM). Accession numbers: AM F125553 (holotype); AM F15818 (referred). All measurement data obtained during this study is contained in the relevant description sections of the specimens.

### New Species Registration

The following information was supplied regarding the registration of a newly described species: Publication LSID: urn:lsid:zoobank.org:pub:C38C6FDF-A1DC-4D39-9F54-8D3A4A866255 Isisfordia molnari sp. nov. LSID: urn:lsid:zoobank.org:act:430A065D-D1F1-459A-8984-1C43D2196FDC.
